# Art boxes supporting parents and infants to share creative interactions at home: an art-based response to improve well-being during COVID-19 restrictions

**DOI:** 10.1016/j.puhe.2021.01.031

**Published:** 2021-04

**Authors:** V.G. Armstrong, J. Ross

**Affiliations:** University of Dundee, UK

**Keywords:** Participative arts, Parent-infant, Arts in health, Well-being, Attachments, Connection

## Abstract

**Objectives:**

This article seeks to demonstrate the impact of distributing boxes of art resources and guided activities for vulnerable parents and infants to do together at home.

**Study design:**

Designed in conjunction with the local arts centre and the psychology team at the University of Dundee, the art boxes were a response to planned face-to-face art interventions with families being cancelled due to COVID-19 restrictions. The aim of the art boxes is to encourage parents to make art together with their infants, fostering connection through playful, creative shared experiences. This research is currently being expanded to reach out to new families through referrals from health visitors, family nurses, and charity partners.

**Methods:**

Data is being collected on how the art boxes are experienced by families using a mixed-methods approach. Families complete feedback cards (online, or using the stamped addressed card included in the box) rating their experience on quantitative scales and providing open comments. Visual data are gathered through parents sharing images with us on social media. An initial sample of 10 participants has been interviewed using semistructured interviews, allowing more in-depth qualitative understanding of their experiences. These preliminary findings are discussed here.

**Results:**

The thematic analysis of initial interviews provided a rich picture of the disconnection families experienced during lockdown, why art boxes may be beneficial to parental well-being, and the mechanisms by which the boxes may help to develop connections for the parent and infant together.

**Conclusions:**

Preliminary findings show parents reporting feeling more confident and undertaking new activities which they plan to continue. This was of particular importance during lockdown where parents report opportunities for different experiences being more limited. Parent’s describe positive playful interactions and reported improvements to their own well-being from doing creative activities together with their child. Analysis of these initial interviews gives a framework of barriers and supports to connection which highlights how art boxes can facilitate connectedness between dyads with the potential to strengthen attachments.

## Highlights

• The Art at the Start project provided an art-based intervention for parents and young children at home.
• We undertook thematic analysis of parent interviews about their experiences.
• This found themes of ways that art making supported connectedness.
• We found evidence of art making improving well-being and supporting connections.

There is a developing case for the social benefits of art,^1^ including the impact of arts on mental health and on the well-being of children. There are positive evaluations of participative arts in the early years^2^ and research shows dyadic art therapy sessions can improve parental well-being and children’s attachments.^3^ However, we know that social factors impact upon arts participation^4^ and in the light of the pandemic existing inequalities have been exacerbated,^5^ as well as parental mental health difficulties).^6^

**Fig. 1:**
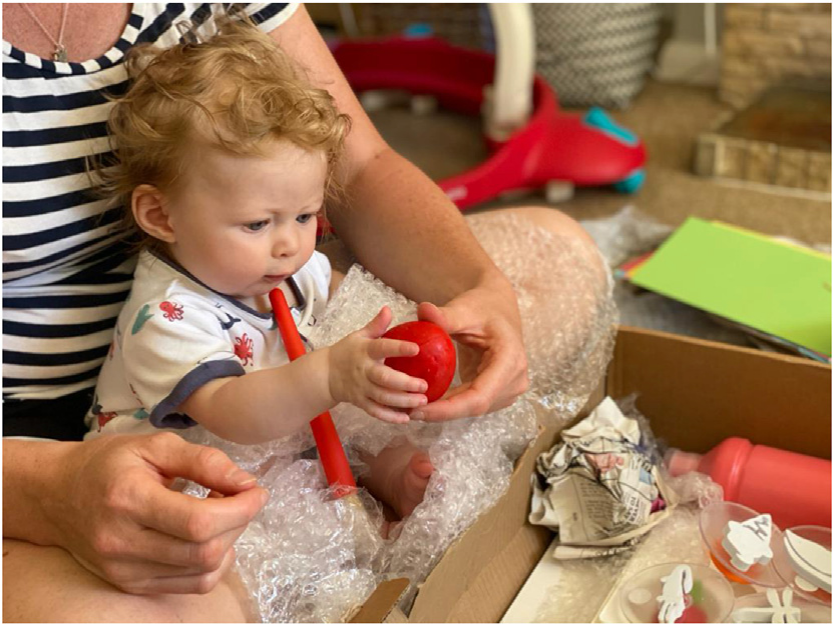
A mother and infant exploring the contents of their art box together.

The Art at the Start project, a collaboration between University of Dundee and Dundee Contemporary Arts, has been developing a family programme of early years participative arts activities, including art therapy sessions targeting families of infants aged 0–3 years. These art therapy sessions take referrals from health and voluntary sector agencies, based on concerns that parents are vulnerable to low well-being and mental ill-health and infants may be at risk of attachment difficulties. When COVID-19 restrictions came into place, several planned art therapy groups were cancelled, affecting more than 40 families. We were concerned about the withdrawal of support for those families and their likely lack of resources to participate in some of the online activities being offered by ourselves and others over this time. To address this concern, we developed an innovative idea to maintain the families’ engagement in joint art making. The art therapist produced boxes containing everything families needed to participate in 12 suggested activities (with variation for age and stage), including information on why these kinds of activities are beneficial. Boxes were sent during the highest restrictions in May 2020, by courier to avoid unnecessary risk from contact. Although these were not a replacement for art therapy, the activities were informed by what we have learnt in art therapy sessions about maximising connections. Activities were designed to encourage parents to make art together at home with their infants, fostering connection through playful, creative shared experiences(Fig. 1). With positive feedback from partner agencies and ongoing need, we were funded to roll out this scheme to reach at least 100 more families who we had not previously been involved with, between June and September when many restrictions remained. We have taken a mixed methods approach to collecting data including postcards with scaling questions in every box and gathering visual data by inviting families to share images through social media. This report focuses on our initial findings from semistructured interviews, conducted by phone, with a sample (*n* = 10) of parents involved, all of whom had their boxes for two months or more to allow time to take part in the activities. Our data provide promising insights into the mechanism by which this scheme may benefit parent-infant relationships.

## Themes from interviews

Semistructured interviewing was chosen to give consistency whilst allowing some freedom to ensure that parents had understood the questions or to ask for further clarification or detail. Thematic analysis was undertaken on the transcripts of interviews using a reflexive model of practice.^7^ In our analysis, we found a framework around barriers and supports to connectedness was a useful way of meaningfully describing all the data. A summary of our overarching themes and subthemes can be seen in Table 1.

**Table 1:**
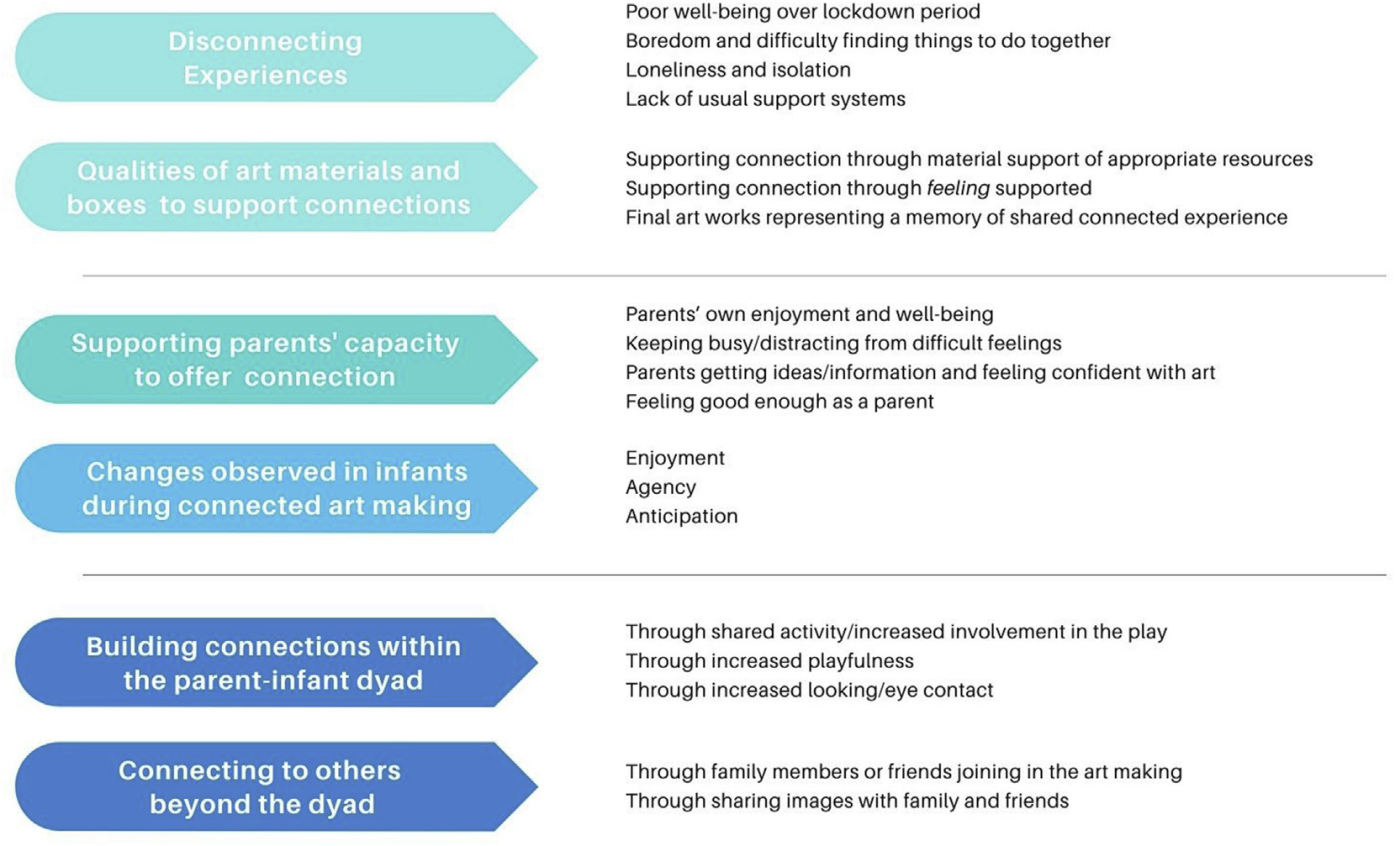
Changes to connection through shared art making at home.

### Disconnecting experiences

All parents reported finding the lockdown period hard and it was clear this had impacted upon their well-being. Parents described the challenge of keeping occupied at home without their normal groups and social activities and the unavailability of shops. Some mentioned the challenge of occupying different ages of children. They reported feeling bored themselves alongside guilt about their infant's experiences. Parents also made statements that specifically reflected loneliness and isolation over this time. In particular, several mentioned not having ‘mum friends’ as they had not had any opportunity to make them. Connected to that feeling of isolation, a couple mentioned how different they felt their experiences had been to their peer groups without children. All parents reported the lack of supports available to them, with the withdrawal of parent support groups and home visits.

### Qualities of art materials and boxes to support connected experiences

All parents reported appreciating the resources and that they would not have had them available without the packs, with reasons including unavailability of shops, not knowing where to go or what would be safe for their child. Having a resource which is age appropriate and available is important if we are to facilitate this kind of play. We asked about activity preferences and responses were broad with the largest number choosing paint. A particular point that was emphasised by the parents was the tactile quality of the materials and how these were appreciated by their infants. We noticed a repeated description from parents of the experience of actually opening up the box itself and exploring the contents with their infants. Other statements framed the art box as a gift. We think this represents the fact that the art boxes become a physical symbol that someone is offering support and that they are ‘held in mind’. One parent made this explicit saying that they felt ‘blessed’ and that they were thought of. The final way in which the physical qualities of art making seemed to support connection was by providing a concrete reminder of positive moments. Parents talked about having art works up on display and of particular things that they would keep, such as clay handprints.

### Supporting parents’ capacity to offer connected experiences

To offer positive connected moments to their child a parent needs to have sufficient emotional availability. We found a number of themes about ways boxes supported the parents so that they in turn could connect to their infants. Parents reported positive feelings themselves from doing activities, including increased happiness, calm, fun and relaxation. Several mentioned their own enjoyment of the art making and others that the boxes had arrived at a good time when they were low. Parents appreciating having something planned that would fill their day. They reported liking to have a focus and that they felt less bored. Some also described how this was able to take their mind off difficulties. A theme emerged of parents feeling guilty about what they were doing with their infants and the art activities helping them to feel they were doing a good enough job.

There was also a practical aspect of parents getting ideas about the kinds of activities they could do and a prompt to do them. They all felt they had needed the instruction booklet, particularly for guidance around what works for younger ages and knowing what was safe. All parents reported feeling more confident from using the box and several described ways in which they were now able to adapt activities to suit or come up with ideas. Previously only three of the parents reported a limited amount of art making but following the box all stated that they would keep making art, some having already accessed online resources from the gallery.

### Changes observed in the infant during connected art making

We asked about changes that parents had seen in their infants and identified three themes reflecting psychological changes for the infant. Parents described signs of enjoyment, that infants were happy, having fun and livelier. Parents also described behaviours that we would consider signs of agency – infants enjoying the consequences of their actions with the materials. For example, moving paint about on the paper, making choices or even getting art materials out for themselves. Parents also thought infants knew what was coming and were looking forward to it. This shows that infants were anticipating the activity, and it held positive associations for them.

### Building connectedness within the dyad

All the parents reported feeling connected to their infant during the art activities and gave interesting insights about what aspects of the art making process might be supporting this connection. All talked about how the art activity was something shared, even that it was something that was meant to be done jointly. They reported being more involved, physically and emotionally, than when doing an equivalent activity, such as playing with toys. An increased amount of participation in play from parents will increase the opportunity for the dyad to have moments of positive connection. Parents also described being more playful with their infant during the art, similarly increasing potential for positive connected experiences. The strongest evidence for psychological connection was premised on eye contact, with parents describing their infants turning to look at them during art making, showing them materials and seeking eye contact. Parents reported responding to these connection seeking opportunities and their own positive feelings arising from them.

### Connecting to others beyond the dyad

A final theme, surprising to us, was how the art boxes had helped the parent-infant dyads to connect with others. Half described family members or friends joining in with their art activity and that this had been a positive thing to share, even decreasing difficult sibling interactions. While not all parents had people around to become involved, they all brought up other ways in which they had shared art works with other people, either by giving physical artworks as gifts or by sharing images over social media. This seemed to have been particularly valued, perhaps because separated from those connections over lockdown.

## Discussion

It seems clear that families used and valued the art boxes and increased their involvement in shared creative activities. Most interesting to us, is the insight into potential mechanisms by which the art boxes might be able to facilitate positive connected experiences for infants and their caregivers. The boxes supported parents so that their own well-being improved, potentially making them more available to their infant,^8^ as well as giving those parents a feeling that they were doing a ‘good enough’ job for their infant and some relief that they had activity planned in their day. The process of art making itself seemed to promote connected experiences by encouraging playful, shared engagement in the activity. This was evidenced in the observation of increased eye contact. These kinds of connected interpersonal experiences between infant and caregiver are what help to build positive attachments^9^^,^^10^ so could offer broad benefits to these families. We do not suggest that these packs could provide the equivalent level of benefit to a face-to-face service such as parent-infant art therapy^3^ without the additional benefits of the therapists support for interactions and containment of difficult emotions. However, in the current circumstances these kinds of interventions could offer a useful resource to improve wellbeing during social distancing measures, where families do not have access to their usual activities, resources and social supports. As social distancing requirements are gradually relaxed, and public spaces reopen, this model of boxes may offer a potential mechanism to connect families to the arts centre, who may not typically engage, and encourage them to participate in this community asset offering free, creative, family activity. Our study will continue to interview participants using these preliminary themes as a model, as well as analysing the data from quantitative feedback and using visual data in the form of images from parents to add insight about those aspects which they found important.

## Author statements

### Acknowledgements

The development of the art boxes was undertaken by the art therapist and first author in consultation with Sarah Derrick, Dundee Contemporary Arts. The authors would like to thank the charities and health partners who made referrals, and in some cases distributed boxes to families, and all the parents and children for being willing to take part and for their amazing creativity at a difficult time.

### Ethical approval

The study received ethical approval from the University of Dundee Ethics Committee.

### Funding

This research was funded in initial stage through the first author’s University of Dundee, School of Social Sciences PhD studentship, and in the second stage of distribution by a University of Dundee, Public Engagement Seed Fund Award. Neither funding source had involvement in the design, data collection or publication.

### Competing interests

There are no competing interests to declare.
